# Predictive factors for short-term and 3-month outcomes in patients with obstructive sleep apnea and anterior circulation stroke without large artery occlusion

**DOI:** 10.3389/fneur.2025.1663834

**Published:** 2025-11-11

**Authors:** Li Zhang, Yang Feng, Bing-Chuan Xie, Zheng-Qi Xie, Di Wu, Xiao-Yun Liu

**Affiliations:** 1 Department of Neurology, The First Hospital of Hebei Medical University, Shijiazhuang, Hebei, China; 2 Department of Neurology, Hebei Hospital of Xuanwu Hospital Capital Medical University, Shijiazhuang, Hebei, China; 3 Department of Neurology, Xuanwu Hospital, Capital Medical University, Beijing, China; 4 Department of Neurology and China-America Institute of Neuroscience, Xuanwu Hospital, Capital Medical University, Beijing, China

**Keywords:** acute ischemic stroke, functional outcome, obstructive sleep apnea, platelet distribution width, white blood cells

## Abstract

**Background:**

Obstructive sleep apnea (OSA) has been recognized as a potential risk factor for stroke. Among the pathophysiological mechanisms involved in stroke, neuroinflammatory response has gained increasing attention. However, the influence of OSA and inflammation on stroke prognosis remains insufficiently clarified.

**Methods:**

A retrospective analysis was conducted using clinical data from patients diagnosed with acute ischemic stroke (AIS) due to non-Large Artery Occlusion (LAO) in anterior circulation who were admitted between May 2018 and October 2021. Patients were stratified based on documented pre-stroke history of OSA. Multiple linear regression was employed to examine associations among hematological parameters, vascular risk factors, and clinical outcomes of AIS.

**Results:**

No statistically significant differences were observed between the OSA and non-OSA groups in terms of National Institutes of Health Stroke Scale (NIHSS) scores at admission, modified Rankin Scale (mRS) scores at discharge, or mRS scores at the 3-month follow-up (all *p* > 0.05). However, the multiple linear regression analysis revealed that a pre-stroke history of OSA was significantly associated with lower discharge NIHSS scores (*p* = 0.005), lower discharge mRS scores (*p* = 0.001), and lower 3-month mRS scores (*p* = 0.004). Platelet distribution width (PDW) was positively associated with higher discharge mRS scores (*p* = 0.018) and 3-month mRS scores (*p* = 0.006). Elevated white blood cell (WBC) counts were positively associated with discharge NIHSS (*p* = 0.001), discharge mRS (*p* < 0.001), and 3-month mRS (*p* = 0.001).

**Conclusion:**

Lower WBC count and the presence of a pre-stroke OSA history may be associated with less severe stroke presentation and more favorable functional outcomes both at discharge and at 3 months post-stroke. Additionally, a lower PDW might associated with better short-term and 3-month outcomes. In cases of anterior circulation AIS without LAO, our findings did not reveal an association between pre-stroke history of OSA with increased stroke severity or poorer prognosis.

## 1 Introduction

Stroke remains a leading cause of mortality globally and contributes significantly to long-term disability and healthcare expenditure (1). In China, over 2.4 million new cases of stroke are reported annually, with mortality reaching 22.3%, and approximately 87% of these cases are classified as ischemic strokes (2, 3). Early evaluation of stroke severity and identification of modifiable risk factors are essential for improving clinical outcomes among patients with stroke.

Obstructive sleep apnea (OSA), a common form of sleep-disordered breathing, has been recognized as a potential risk factor for stroke. OSA is characterized by recurrent pharyngeal collapse during sleep, and its contribution to cerebrovascular risk is associated with several mechanisms, including oxidative stress, release of pro-inflammatory and pro-thrombotic mediators, glucose intolerance, fluctuations in cerebral blood flow, nocturnal hypoxemia, and increased sympathetic nervous system activity and blood pressure. Among patients with stroke, OSA has been associated with poorer functional outcomes at 3 and 12 months, prolonged hospitalization and rehabilitation, increased recurrence rates, and higher mortality (4, 5). However, findings from a cohort of 41,742 Swedish adults indicated that a pre-stroke history of snoring or transient apneic episodes was not associated with an increased risk of ischemic stroke (6). The relationship between OSA and stroke appears to be bidirectional, with OSA contributing both as a predisposing factor for stroke and as a complication following stroke onset (7). Therefore, OSA may exert different pathophysiological effects depending on its timing in relation to the occurrence of acute ischemic stroke (AIS). Beyond its role as a respiratory disorder, OSA is increasingly recognized as a systemic condition linked to metabolic and vascular comorbidities. Previous evidence has demonstrated that anthropometric measurements in OSA patients also can serve as valuable predictors of cardiometabolic diseases (8).

Neuroinflammation represents a critical pathophysiological mechanism in AIS (9, 10). The inflammatory response disrupts the blood–brain barrier through oxidative stress and activation of matrix metalloproteinases, which intensifies ischemic injury and promotes neuronal death within the ischemic penumbra (11).

Currently, limited data are available regarding prognostic indicators among patients with OSA who experience anterior circulation cerebral infarction without large artery occlusion (LAO). The aim of this present study was to evaluate factors influencing short-term and 3-month clinical outcomes in patients with AIS and pre-existing OSA, focusing on hematological parameters, medical history, and clinical characteristics. Unlike most previous studies that assessed OSA following AIS, the current analysis included only patients with a pre-stroke diagnosis of OSA, thereby offering additional insight into its prognostic relevance. These findings may contribute to a better understanding of AIS pathophysiology and support the development of individualized therapeutic and preventive strategies.

## 2 Materials and methods

### 2.1 Study participants

Adult patients (> 18 years) with first-onset anterior circulation AIS, specifically those without culprit large artery (internal carotid artery, middle cerebral artery, anterior cerebral artery) occlusion, who were consecutively admitted to the Department of Neurology at Xuanwu Hospital from May 2018 to October 2021 were included in this study. Stroke severity and functional outcomes were evaluated using the National Institutes of Health Stroke Scale (NIHSS) and modified Rankin Scale (mRS) within 3 days of symptom onset and during follow-up. “Acute” was defined as symptom onset within 72 h prior to hospital admission. Patients were divided into OSA and non-OSA groups based on pre-stroke medical history. During the AIS phase, all patients received care in accordance with the Chinese Guidelines for Diagnosis and Treatment of AIS. Cranial magnetic resonance imaging (MRI) was performed within 7 days of onset using a 3.0 T MRI scanner to confirm anterior circulation AIS.

Patients with a previous history of stroke and those lacking sufficient clinical or radiological data were excluded. The following additional exclusion criteria were applied: (1) cerebral hemorrhage, intracranial tumor, demyelinating disease, central nervous system inflammatory disease, history of craniotomy, history of major head trauma, idiopathic intracranial hypertension, aneurysm, arteriovenous fistula, or multiple sclerosis; (2) baseline oxygen saturation < 90%, tracheotomy, current mechanical ventilation, or other positive airway pressure ventilation for therapeutic purposes; (3) acute or chronic pulmonary diseases (such as chronic obstructive pulmonary disease, bronchitis, interstitial pneumonia, pulmonary infection), acute myocardial infarction, other severe cardiopulmonary organ dysfunction, malignancy, hypothyroidism; (4) recent surgery involving the upper airway; (5) neuromuscular junction disease; (6) neurodegenerative diseases such as Parkinson’s disease, motor neuron disease; (7) other sleep disorders such as insomnia, restless legs syndrome; (8) pregnancy; (9) intracranial chronic lesions visible on initial T2-weighted imaging; (10) mRS ≥ 1 point prior to AIS.

NIHSS was used to evaluate stroke severity at admission and discharge. The mRS was used to assess functional outcome at discharge and at the 3-month stroke follow-up after discharge. A good outcome at 3 months post-onset was defined as an mRS of 0 to 2. AIS was classified according to etiology based on the Trial of Org 10,172 in Acute Stroke Treatment (TOAST) criteria, which categorize ischemic stroke into five types: Adams et al. (12) large artery atherosclerosis, cardioembolism, small vessel occlusion, other determined etiology, and undetermined etiology. Baseline demographic variables and vascular risk factors were recorded, including hypertension, diabetes mellitus, coronary artery disease, hyperlipidemia, atrial fibrillation, transient ischemic attack, hyperuricemia, hyperhomocysteinemia, alcohol consumption, and smoking. Upon admission, assessments included blood pressure, complete blood count, fasting blood glucose, glycated hemoglobin levels, blood lipids, electrocardiogram, carotid duplex ultrasound, transcranial Doppler, 24-h Holter monitor, two-dimensional echocardiography, and either head and neck computed tomography (CT) angiography or magnetic resonance angiography (MRA). In some cases, transesophageal echocardiography and cerebral angiography were also performed. Body mass index (BMI) was calculated as weight (kg)/height (m)^2^.

The OSA group included patients with pre-AIS apnea hypopnea index (AHI) ≥ 15 who had not received treatment for OSA. All had completed at least one overnight polysomnography before AIS onset. According to evidence, an AHI ≥ 15 is associated with increased risk of cardiovascular and cerebrovascular events (13–15). The non-OSA group included patients who did not snore or snored occasionally (1 or 2 nights per week). Sleep habits and OSA risk were assessed through patient and cohabiting relative responses to three validated questionnaires: the Berlin Apnea Questionnaire, STOP-BANG Questionnaire, and Epworth Sleepiness Scale (ESS), all completed based on pre-stroke conditions. Patients identified as low risk for OSA were assigned to the non-OSA group. The Berlin Questionnaire includes 10 items and BMI and blood pressure information, evaluating three domains: snoring and apnea, excessive daytime sleepiness, and BMI with hypertension. Absence or only one positive score indicates low OSA risk (16). The STOP-BANG questionnaire consists of 8 dichotomous (yes/no) questions on snoring, tiredness, observed apnea, hypertension, BMI, age, neck circumference, and sex (17). Scores of 0 to 2 indicate low OSA risk. The ESS, developed to assess daytime sleepiness risk, is also used to identify OSA; a score ≥ 9 is considered clinically significant (18).

Studies indicate that STOP-BANG scores ≥ 3 yield sensitivities of 93 and 100% for detecting moderate to severe OSA (AHI > 15) and severe OSA (AHI > 30), respectively, with negative predictive values of 90 and 100%. This makes STOP-BANG a practical tool for high-risk screening. The Berlin Questionnaire also demonstrates good sensitivity for AHI > 15, while the ESS has high specificity across all OSA severities (19). To maximize both sensitivity and specificity, all three instruments were applied. Given that self-reported sleep behavior may underestimate snoring frequency, partner interviews were included. Individuals were excluded from the study if reliable information from relatives regarding sleep habits was unavailable.

### 2.2 Statistical analysis

All analyses were performed using SPSS 26.0 (SPSS Inc.). Continuous data with normal distribution were presented as mean ± standard deviation, and those with non-normal distribution as median and interquartile range (IQR). Categorical data were expressed as percentages. Between-group comparisons of continuous data were performed using independent sample *t*-tests (for normally distributed data) or Mann–Whitney U tests (for non-normally distributed data). Chi-squared tests were used for categorical variables.

Multiple linear regression analysis was performed to evaluate the associations between stroke severity and functional outcomes and various predictors, including OSA, hematological parameters, and other cerebrovascular disease-related factors. NIHSS at discharge and mRS at discharge and at 3-month follow-up were used as dependent variables. Independent variables included demographic factors (sex, age, BMI); medical history variables (hypertension, diabetes mellitus, transient ischemic attack, hyperlipidemia, hyperhomocysteinemia, coronary heart disease, atrial fibrillation, hyperuricemia); OSA grouping (OSA or non-OSA). Behavioral factors such as smoking and alcohol consumption, as well as thrombolysis status were also included. Hematological parameters comprised red blood cells, hematocrit, white blood cells (WBC), lymphocytes, eosinophils, platelets, mean platelet volume (MPV), platelet distribution width (PDW), red cell distribution width, neutrophils (NEU), monocytes, albumin, D-dimer, and plasma fibrinogen. Additionally, the following composite inflammatory markers were analyzed: neutrophil-to-lymphocyte ratio (NLR), platelet-to-lymphocyte ratio (PLR), monocyte-to-lymphocyte ratio (MLR), monocyte-to-high density lipoprotein cholesterol ratio (MHR), systemic immune-inflammation index (SII, neutrophils × platelets count/lymphocytes), coagulation-inflammation index (SCI, platelets count × fibrinogen/WBC), and systemic inflammation response index (SIRI, neutrophils count × monocytes count/lymphocytes count). A *p* value < 0.05 (two-tailed test) was considered statistically significant.

## 3 Results

### 3.1 Overall baseline data

A total of 82 patients with AIS met all the inclusion criteria. Among these, 31 were assigned to the OSA group and 51 to the non-OSA group. The overall prevalence of OSA, defined as AHI ≥ 15, was 37.8% (28 males, 3 females). Polysomnography data were available for 21 patients in the OSA group, with AHI values ranging from 16.10 to 59.50 (median = 35.60). Baseline oxygen saturation (SpO_2_) ranged from 87 to 97% (median = 93.6%), and minimum SpO_2_ ranged from 30 to 84% (median = 76%). Patient demographic and clinical characteristics are presented in Table 1. Patient age ranged from 30 to 86 years (mean = 58.27 years), and males accounted for 76.83% of the cohort.

**Table 1:** Baseline clinical and demographic characteristics of patients with AIS.

Clinical characteristics	Non-OSA group (*N* = 51)	OSA group, AHI ≥ 15 (*N* = 31)	*p* value^a^
Age, *x̄* (S)	59.92 (14.15)	55.55 (11.80)	0.153
Male, *N* (%)	35 (68.63)	28 (90.32)	0.024
BMI, M (IQR)	24.22 (3.44)	26.23 (4.50)	0.001
Risk factors, *N* (%)			
Hypertension	35 (68.63)	25 (80.65)	0.234
Diabetes mellitus	15 (29.41)	19 (61.29)	0.004
Hyperlipidemia	28 (54.90)	15 (48.39)	0.567
Coronary heart disease	7 (13.73)	5 (16.13)	1.000
Atrial fibrillation	7 (13.73)	4 (12.90)	1.000
Transient ischemic attack	12 (23.53)	5 (16.13)	0.423
Hyperuricemia	3 (5.88)	1 (3.23)	0.990
Hyperhomocysteinemia	9 (17.65)	6 (19.35)	0.846
Smoking history	21 (41.18)	19 (61.29)	0.077
Drinking history	13 (25.49)	10 (32.26)	0.508
Thrombolysis, *N* (%)	9 (17.65)	3 (9.68)	0.504
TOAST classification, *N* (%)			
Large artery atherosclerosis	22 (43.14)	9 (29.03)	0.202
Cardioembolism	5 (9.80)	3 (9.68)	1.000
Small vessel occlusion	22 (43.14)	15 (48.39)	0.643
Other determined etiology	0 (0)	2 (6.45)	0.272
Undetermined etiology	2 (3.92)	2 (6.45)	1.000
Hematological parameters			
Red blood cell, *x̄* (s)	4.59 (0.45)	4.80 (0.65)	0.088
Hematocrit, *x̄* (s)	41.30 (3.75)	42.69 (5.15)	0.167
White blood cell, *x̄* (s)	6.62 (1.66)	7.81 (2.23)	0.008
Neutrophil, *x̄* (s)	4.46 (1.54)	5.41 (2.09)	0.035
Monocyte, M (IQR)	0.41 (0.22)	0.48 (0.16)	0.009
Lymphocyte, *x̄* (s)	1.81 (0.62)	1.94 (0.50)	0.337
Eosinophil, M (IQR)	0.09 (0.14)	0.16 (0.14)	0.075
Platelet, *x̄* (s)	210.86 (52.97)	226.10 (48.31)	0.202
MPV, M (IQR)	10.90 (1.10)	10.95 (1.10)	0.637
PDW, *x̄* (s)	12.61 (1.81)	12.78 (1.72)	0.370
RDW, M(IQR)	12.80 (0.90)	12.45 (1.15)	0.243
Albumin, *x̄* (s)	39.29 (3.69)	40.52 (4.68)	0.196
D-dimer, M (IQR)	0.32 (0.47)	0.32 (0.48)	0.845
Fibrinogen, M (IQR)	3.20 (0.93)	3.50 (1.06)	0.005
NLR, M (IQR)	2.51 (1.55)	2.60 (1.40)	0.261
PLR, M (IQR)	110.30 (63.91)	106.65 (56.64)	0.687
MLR, M (IQR)	0.23 (0.12)	0.26 (0.12)	0.081
MHR, M (IQR)	0.33 (0.21)	0.43 (0.23)	0.025
SII, M (IQR)	455.56 (436.47)	576.44 (494.02)	0.207
SCI, M (IQR)	103.67 (44.14)	108.89 (60.05)	0.415
SIRI, M (IQR)	1.05 (0.86)	1.17 (1.37)	0.044
Sleep characteristics, median (range)			
AHI	—	35.60 (16.10 ~ 59.50)	—
Baseline SpO_2_	—	93.6% (87 ~ 97%)	—
Minimum SpO_2_	—	76% (30 ~ 84%)	—

OSA, obstructive sleep apnea; S, standard deviation; M, median; IQR, interquartile range; BMI, body mass index; TOAST, Trial of Org 10,172 in Acute Stroke Treatment; MRI, magnetic resonance imaging; MPV, mean platelet volume; PDW, platelet distribution width; RDW, red cell distribution width; NLR, neutrophil-to-lymphocyte ratio; PLR, platelet-to-lymphocyte ratio; MLR, monocyte-to-lymphocyte ratio; MHR, monocyte-to-high density lipoprotein cholesterol ratio; SII = neutrophils × platelets count/lymphocytes; SCI = platelets count × fibrinogen/WBC; SIRI = neutrophils count × monocytes count/lymphocytes count; AHI, apnea-hypopnea index; SpO_2_, oxygen saturation.

a *p* values: values for continuous normally distributed variables are from independent-samples *t* tests; for non-normally distributed variables, Mann–Whitney U tests were used; categorical variables were compared using the chi-square (*χ*^2^) test.

The OSA group exhibited a significantly higher proportion of male patients (90.32%) compared to the non-OSA group (68.63%) (*p* = 0.024). BMI was also significantly higher in the OSA group (26.23) compared to the non-OSA group (24.22) (*p* = 0.001). Additionally, the proportion of patients with diabetes mellitus was significantly greater in the OSA group (61.29%) than in the non-OSA group (29.41%) (*p* = 0.004). Compared to the non-OSA group, the OSA group demonstrated significantly higher levels of WBC (*p* = 0.008), neutrophils (*p* = 0.035), monocytes (*p* = 0.009), MHR (*p* = 0.025), fibrinogen (*p* = 0.005), and SIRI (*p* = 0.044).

### 3.2 Differences in NIHSS and mRS between groups

No statistically significant differences were observed between the OSA and non-OSA groups in terms of NIHSS scores or mRS scores. Specifically, admission NIHSS was 4 [interquartile range (IQR) = 6] in the non-OSA group and 3 (IQR = 6) in the OSA group (*p* = 0.384). Discharge NIHSS was 3 (IQR = 4) for the non-OSA group and 1 (IQR = 5) for the OSA group (*p* = 0.226). Discharge mRS scores were 2 (IQR = 3) in the non-OSA group and 1 (IQR = 2) in the OSA group (*p* = 0.247). At the 3-month follow-up, mRS scores remained comparable between groups [non-OSA: 1 (IQR = 1); OSA: 1 (IQR = 2); *p* = 0.365] (Table 2).

**Table 2:** Neurological severity and functional outcome measures in patients with AIS.

Clinical characteristics	Non-OSA group (*N* = 51)	OSA group, AHI ≥ 15 (*N* = 31)	*p* value^a^
NIHSS score at admission, M (IQR)	4 (6)	3 (6)	0.384
NIHSS score at discharge, *N* (%)			
≤5	39 (76.47)	25 (80.65)	0.658
6–10	10 (19.61)	4 (12.90)	0.434
>10	2 (3.92)	2 (6.45)	1.000
NIHSS score at discharge, M (IQR)	3 (4)	1 (5)	0.226
mRS score at discharge ≤ 2 months, *N* (%)	34 (66.67)	21 (67.74)	0.920
mRS score at discharge, M (IQR)	2 (3)	1 (2)	0.247
mRS score ≤ 2 at 3 months, *N* (%)	45 (88.24)	27 (87.10)	1.000
mRS score at 3 months, M (IQR)	1 (1)	1 (2)	0.365

a *p* values: for continuous variables with normal distribution, comparisons were conducted using the independent samples *t*-test; for non-normally distributed continuous variables, the Mann–Whitney U test was used; and for categorical variables, the chi-squared (*χ*^2^) test was applied. M, median; IQR, interquartile range; NIHSS, National Institutes of Health Stroke Scale; mRS, modified Rankin Scale.

### 3.3 Multiple linear regression analysis results

White blood cell count was positively associated with discharge NIHSS [*p* = 0.001; 95% confidence interval (CI) = 0.072 to 0.254], discharge mRS (*p* < 0.001; 95% CI = 0.022 to 0.073), and 3-month mRS (*p* = 0.001; 95% CI = −0.600 to 0.112). A history of pre-stroke OSA was negatively associated with discharge NIHSS (*p* = 0.005; 95% CI = −0.906 to 0.168), discharge mRS (*p* = 0.001; 95% CI = −0.285 to −0.076), and 3-month mRS (*p* = 0.004; 95% CI = −0.234 to −0.045). PDW was positively associated with discharge mRS (*p* = 0.018; 95% CI = 0.006 to 0.063) and 3-month mRS (*p* = 0.006; 95% CI = 0.011 to 0.062) (Table 3).

**Table 3:** Multiple linear regression analysis of factors associated with stroke severity and functional outcomes in AIS.

Dependent variable	Independent variable	*β*	*t*	*p*	β 95% CI	
					Lower	Upper
NIHSS score at discharge	White blood cells	0.163	3.579	0.001	0.072	0.254
	Pre-stroke OSA history	−0.537	−2.903	0.005	−0.906	−0.168
	History of atrial fibrillation	−0.504	−2.044	0.045	−0.995	−0.013
mRS score at discharge	White blood cells	0.048	3.755	<0.001	0.022	0.073
	Pre-stroke OSA history	−0.181	−3.437	0.001	−0.285	−0.076
	Thrombolysis	−0.180	−2.564	0.012	−0.320	−0.040
	PDW	0.035	2.417	0.018	0.006	0.063
mRS score at 3 months	White blood cells	0.041	3.601	0.001	−0.600	0.112
	Thrombolysis	−0.196	−3.107	0.003	−0.322	−0.070
	Pre-stroke OSA history	−0.140	−2.956	0.004	−0.234	−0.045
	PDW	0.036	2.830	0.006	0.011	0.062

OSA, obstructive sleep apnea; NIHSS, National Institutes of Health Stroke Scale score; mRS, modified Rankin Scale; PDW, platelet distribution width.

## 4 Discussion

OSA has been associated with increased stroke risk, both indirectly through its influence on vascular disease risk factors and directly as an independent contributor. The pathophysiological features of OSA, including intermittent hypoxemia, sleep fragmentation, increased sympathetic nervous system activity, oxidative stress, systemic inflammation, hypercoagulability, cerebral blood flow alterations, insulin resistance, glucose metabolism abnormalities, and endothelial dysfunction, may affect functional recovery following stroke (20). Additionally, OSA may influence outcomes by increasing the burden of cardiovascular and cerebrovascular risk factors associated with stroke. In contrast to earlier studies, the present findings indicated no significant differences in stroke severity or functional prognosis between patients with and without OSA. After adjustment for confounding variables using multiple linear regression, a history of pre-stroke OSA may be associated with more favorable outcomes, as indicated by lower NIHSS at discharge and lower mRS scores at both discharge and 3 months. This study focused on the impact of OSA diagnosed prior to the first episode of AIS, thereby reducing the possibility that cerebrovascular disease may have contributed to the onset of OSA.

When diagnosing OSA, PSG is the gold standard diagnostic method, and its application in sleep laboratories is of vital importance. However, in actual clinical practice, especially for patients with AIS, relying entirely on polysomnographic monitoring is often impractical. If, in this study, we had insisted on using the same complete polysomnographic monitoring method as in previous prospective studies, a large number of subjects who originally met the study criteria would have been excluded due to their inability to undergo monitoring.

For patients who have not undergone polysomnographic monitoring but are at high risk for OSA, an appropriate screening method is particularly crucial. It can not only help doctors or sleep specialists identify potential OSA patients at an early stage but also improve the efficiency and accuracy of diagnosis. Therefore, in this retrospective study, we employed a brief and precise detection tool—questionnaire assessment—to screen patients in the non-OSA group. Specifically, we included patients who were assessed as having a low risk of OSA by the Berlin Questionnaire and STOP-Bang Questionnaire and had an Epworth Sleepiness Scale (ESS) score of less than 9. This method can effectively identify patients at low risk of OSA and, to some extent, compensate for the limitations of polysomnographic monitoring in practical applications, providing strong support for clinical diagnosis.

Previous studies may have been affected by reverse causality, with OSA resulting from, rather than contributing to, stroke. To current knowledge, this is the first study to report that, among patients with anterior circulation AIS and no culprit LAO, a history of OSA did not result in worse prognosis. Pre-stroke OSA may be a negative predictor of stroke severity and functional outcome at both discharge and 3-month follow-up. Although OSA has been associated with increased incidence of stroke, the current analysis does not support an association with worse outcomes following stroke onset. These findings align with results reported by Lapow et al. (21), which demonstrated that OSA was independently associated with lower in-hospital mortality, reduced intracranial hemorrhage, and decreased rates of hydrocephalus after thrombectomy. Despite the higher prevalence of diabetes mellitus and obesity among patients with OSA, these individuals did not exhibit worse outcomes, potentially due to the neuroprotective effects of ischemic preconditioning. Repeated episodes of intermittent hypoxia and hypercapnia during sleep in patients with OSA may promote adaptive mechanisms that enhance tolerance to ischemic injury. This phenomenon, referred to as ischemic preconditioning, is believed to upregulate genes involved in angiogenesis, promote cerebral vascular remodeling, and enhance perfusion capacity. Research has demonstrated that gene expression related to angiogenesis is elevated 24 h following hypoxic–ischemic preconditioning, which may contribute to improved cerebral blood flow in individuals with intracranial arterial stenosis (22–24). These mechanisms may account for improved cerebrovascular reserve and favorable outcomes in some patients with OSA. Furthermore, the results of this study may also be influenced by the obesity paradox. Obesity is a risk factor for many cardiovascular and cerebrovascular diseases. However, obese patients have higher fat tissue content, which leads to an increase in the secretion of chemical mediators (such as tumor necrosis factor-*α* receptor), thereby reducing the inflammatory response after a stroke (25). The obesity paradox is a phenomenon where patients with elevated body mass index in many cardiovascular diseases (including stroke) have lower incidence and mortality rates. Dicpinigaitis et al. (26) also observed the obesity paradox in their study, and the study found that the adjusted mortality rate of obese AIS patients was lower. Our analysis found that the OSA group was more likely to be obese than the non-OSA group.

Inflammatory mechanisms contribute to the onset and progression of AIS through multiple pathways. The interaction between inflammation and thrombosis plays a pivotal role in AIS-related brain injury, with WBC and platelets acting as central mediators in this process (27). The interaction between these cellular components represents a critical interface of inflammatory and thrombotic cascades. WBC may aggravate ischemic injury through several mechanisms (28, 29).

First, peripheral vascular and tissue edema can reduce white blood cell deformability while significantly enhancing adhesion and aggregation. These cells may accumulate and adhere to vascular endothelial surfaces, leading to microcirculatory disturbances and exacerbation of cerebral tissue ischemia and hypoxia. Second, activated WBC generate oxygen free radicals, matrix metalloproteinases, and reactive oxygen species, which can damage vascular endothelial integrity and increase permeability of the blood–brain barrier. Additionally, the release of vasoactive substances by activated WBC promotes vasoconstriction and platelet aggregation, further contributing to neuronal injury. Furlan et al. (30), in an analysis of 8,829 patients enrolled in the Canadian Stroke Network, reported that elevated white blood cell count at admission independently predicted increased disability at discharge (mRS), with a 1.04-fold increase in disability risk per 1 × 10^9^/L increment in white blood cell count. Their findings also demonstrated that higher admission white blood cell counts were associated with increased 30-day case fatality rates. Similar associations were reported by Christensen et al. in 719 patients with AIS, where admission white blood cell count within 24 h correlated with poor clinical prognosis but not with 1-year mortality (31). Zhou et al. (32) reported consistent findings in 325 young patients with AIS, demonstrating that elevated white blood cell counts were significantly associated with stroke severity and poor prognosis at 14 days.

However, previous studies did not differentiate between LAO and non-occlusion, nor between anterior and posterior circulation. The current study demonstrated that higher admission white blood cell counts may be correlated with more severe neurological impairment and worse functional outcomes at discharge and at 3 months in patients with anterior circulation AIS without LAO. These findings provide added significance, as LAO is a known independent determinant of infarct volume and AIS severity. Additionally, anatomical and hemodynamic differences between anterior and posterior circulation, such as vessel diameter, blood flow velocity, shear stress, and compensatory capacity may account for differential vascular vulnerability and injury mechanisms. Exploring the region-specific effects of cerebrovascular risk factors contributes to understanding stroke pathogenesis more comprehensively.

In line with inflammation-based pathophysiology, a variety of composite inflammatory indices have emerged in recent years, including the NLR, PLR, MLR, MHR, SII, SCI, and SIRI. These indices integrate peripheral blood parameters and are generally considered to more reliably reflect systemic inflammatory status than single biomarkers. Their diagnostic and prognostic utility in stroke has gained research interest. However, after adjusting for conventional risk factors and laboratory indicators in the current analysis, none of these composite markers demonstrated significant prognostic value. Further validation in large-scale, prospective studies is warranted to elucidate their potential predictive role.

Platelets, derived from mature megakaryocytes in the bone marrow, play essential roles in coagulation, inflammation, thrombosis, and atherosclerosis. PDW reflects variability in platelet size and is considered a marker of platelet activation. Elevated PDW values indicate greater size heterogeneity and enhanced platelet reactivity. Compared to mean platelet volume, PDW is thought to more directly represent platelet activation (33). Some reports have indicated that PDW is not significantly affected by blood collection or storage times, indicating its potential as a stable biomarker (34). Nonetheless, previous investigations into thromboembolic conditions have largely overlooked PDW as a prognostic indicator.

A case–control study identified PDW as an independent risk factor for early-onset coronary artery disease (35). Sarkar et al. (36) reported that PDW could predict AIS severity, although that study excluded patients with diabetes mellitus and hypertension, and limited its focus to motor dysfunction severity. Xie et al. (37) found that PDW was independently associated with poor outcomes in patients with AIS receiving thrombolytic therapy, though subgroup analyses by occlusion status or circulation territory were not performed. The present study demonstrated that in anterior circulation patients with AIS without LAO, higher PDW may be associated with poorer short-term and 3-month clinical outcomes. These results aligned with findings by Xie et al. (37); however, PDW was not significantly associated with stroke severity as measured by discharge NIHSS in the current analysis. In contrast, Gao et al. (38) reported that lower PDW levels were associated with poor outcomes at 3 months following intravenous thrombolysis in AIS. The inconsistencies across studies may be attributed to differences in study design, patient baseline characteristics, timing of blood sampling, use of anticoagulants, and laboratory equipment, which could introduce heterogeneity. Nevertheless, even in the context of conflicting evidence, the observed association between PDW and AIS outcomes provides a potentially valuable reference point for clinical decision-making.

This study has certain limitations. Firstly, as a single-center study, the population covered by this research is relatively limited and the sample size is small, which may lead to inherent statistical bias and result in skewed outcomes. Secondly, being a retrospective study, some indicators such as white blood cell count and platelet distribution width, among others, were not dynamically monitored during the treatment process, thus lacking data in these aspects and hindering the assessment of their possible association with prognosis. Thirdly, the non-OSA group did not undergo polysomnography before acute ischemic stroke; classification was based solely on three questionnaire-based screening tools. These questionnaires are designed for preliminary screening rather than definitive diagnosis, and therefore cannot conclusively distinguish between OSA and non-OSA patients. Finally, although BMI was included as an independent variable in the multivariate regression analysis to minimize confounding, the potential influence of the obesity paradox on clinical outcomes cannot be fully excluded, and some residual confounding related to body composition and undiagnosed OSA may still exist.

## 5 Conclusion

The findings of this study indicated that lower WBC count and a history of pre-stroke OSA may be associated with reduced stroke severity and more favorable functional outcomes both in the short-term and at 3-month follow-up in patients with anterior circulation AIS without LAO. Additionally, lower PDW may be associated with improved short-term and 3-month functional prognosis in the patients. Due to the high heterogeneity among different articles, the interpretation of the results should be cautious. However, the current results also have certain significance and can provide good references and inspirations for future higher-quality prospective, multicenter cohort studies on AIS. Further investigation through dynamic monitoring and large-scale, multi-center prospective studies is warranted to validate these findings and enhance the evidence base. Such data may contribute to the refinement of clinical risk stratification and therapeutic strategies aimed at improving functional outcomes in post-stroke populations.

## Data Availability

The original contributions presented in the study are included in the article/supplementary material, further inquiries can be directed to the corresponding author.

## Glossary

OSA: obstructive sleep apnea
AIS: acute ischemic stroke
LAO: large artery occlusion
NIHSS: National Institute of Health Stroke Severity Scale
mRS: modified Rankin Scale
PDW: platelet distribution width
WBC: white blood cell
MRI: magnetic resonance imaging
TOAST: Trial of Org 10,172 in Acute Stroke Treatment
CT: computed tomography
BMI: body mass index
AHI: apnea-hypopnea index
ESS: Epworth Sleepiness Scale
IQR: interquartile range
TIA: transient ischemic attack
NEU: neutrophil
NLR: neutrophil-to-lymphocyte ratio
PLR: platelet-to-lymphocyte ratio
MLR: monocyte-to-lymphocyte ratio
MHR: monocyte-to-high density lipoprotein cholesterol ratio
SII: systemic immune-inflammation index
SCI: systemic coagulation-inflammation index
SIRI: systemic inflammatory response index
SpO_2_: saturation of pulse oxygen
MPV: mean platelet volume
RDW: red blood cell distribution width
CI: confidence interval
